# Health related quality of life and its correlates among people with depression attending outpatient department in Ethiopia: a cross sectional study

**DOI:** 10.1186/s12955-019-1233-7

**Published:** 2019-11-08

**Authors:** Seid Shumye, Zelalem Belayneh, Nebiyu Mengistu

**Affiliations:** Department of Psychiatry, College of Health and Medical Science, Dilla University, Dilla, Ethiopia

**Keywords:** Quality of life, Depression, Mental illness, Psychosocial support, Mental disorder

## Abstract

**Background:**

Depression is a common mental disorder negatively affects the cognitive, emotion, behavior, functionality and quality of life of people. Poor quality of life results in high rates of relapse, inability to perform occupational and social activities, impaired future outlook, and increases overall health care related costs. However, there is no available evidence regarding the health related quality of people with depression in Ethiopia. Therefore, evaluating the quality of life of people with depression is crucial.

**Objective:**

The aim of this study was to assess the health related quality of life and its correlates among people with depression at Amanuel Mental Specialized Hospital, Addis Ababa, Ethiopia.

**Methods:**

An institutional based cross-sectional study was conducted from May ^1st^ to ^30th^, 2018. A randomly selected 394 clients with depression were participated in this study. Health related quality of life was measured using world health organization quality of life brief. The collected data were coded and entered to SPSS version 20 for analysis. Step wise multiple linear regression analysis was used to identify the correlates of quality of life and the strength of the correlation was measured by β coefficient with 95% confidence interval.

**Results:**

The mean (±SD) scores of quality of life of people with depression were 41.3 ± 7.5, 42.8 ± 8.2, 38.9 ± 8.9 and 41.8 ± 6.5 for physical, psychological, social and environmental domains, respectively. The Multiple regression analysis showed that age of respondents, age of onset of depression, perceived stigma, living arrangement, social support level and duration of illness were statistically significant predictors of health related quality of life of people with depression in all or at least one domain of quality of life.

**Conclusions:**

This study revealed that nearly half of study participants scored below the mean score in each domain of health related quality of life. This demonstrates a need for improving the quality of life of people with depression through the integration of a positive mental health approach and bio-psychosocial view together with the pharmacological treatments of depression. Moreover, strengthening social support, early identification and treatment of depression and prevention of stigma are also highly recommended to improve the quality of life of people with depression.

## Background

Quality of life (QOL) is defined as “individuals’ perceptions of their position in the context of the culture and value systems they live and in relation to their goals, expectations, standards and concerns” [1, 2]. It is a broad ranging concept incorporating the persons’ physical health, psychological state, level of independence, social relationships, and their relationships to silent features of the environment [2–4]. In recent times, QOL is considered as an important component and prognostic indicator of mental illness and recommended to be integrated in the clinical evaluation and interventions of people with severe mental illness [3, 4].

People with severe mental illness are more vulnerable to have a diminished health related quality of life, and depression takes a higher proportion [4, 5]. Thus, the nature of symptoms (loss of interest, depressed mood, lack of interest for pleasurable activities, low self esteem, psychomotor retardation and other) and its comorbod illnesses together with the social, occupational and cognitive impairments significantly affect the quality of life of people with depression [6, 7]. The prevalence of depression is estimated to be 11% and 9.1% globally and in Ethiopia, respectively [8, 9].

Literatures showed the quality of life of people with depression is highly impaired in both the developed and developing nations [10, 11]. Age of patients, onset of depression, medication non-adherence, comorbod illness, and poor social support, perceived stigma of their depressive status and family history of depression were found to have a statistically significant association with health related quality of life of people with depression [12–15]. The poor quality of life among people with depression again can increase the vulnerability for medical complications such as chronic heart disease, diabetes mellitus and hypertension due to the immunity compromization effects of stress related to poor quality of life [16, 17].

Patients with depression and their relatives increasingly expect improvements not only their symptoms, but beyond that improvements of their functioning and quality of life [18, 19]. There is also a growing consensus that successful treatment of depression should not only target symptom severity, but also impairment in functioning and QOL in leading to restoration of health [20, 21]. The scope of treatment of people with depression therefore, extends to the patients’ subjective feelings of wellbeing, satisfaction, functioning and impairment beyond to the traditional symptoms reductions approaches [3, 21].

Recently, innovative and emerging concepts of euthymia are introduceced to incorporate the psychological construct of health-related quality of life to comprehensive state of positive mental health [22]. This is characterized not simply by the absence of symptoms of depression [23], but by the presence of psychological well-being, quality of life, psychological flexibility, resilience, frustration tolerance and resistance to stress [23–25]. Thus, this is the time that clinical psychologists and other professionals working for mental health related activities have to move from the traditional psychopathology-based perspective to a positive clinical approach to significantly diminish the risk of recurrence of affective disorders, and to use innovative assessment and intervention strategies of health related quality of life [26, 27].

Despite of this considerable opportunity and clinical importance of addressing the quality of life of people with depression, professionals still have a considerable focus only on pharmacotherapy for symptomatic reduction and ignore quality of life of patients with depression [26]. This is the single aspect of treating patients with depression commonly practiced, particularly in developing countries including Ethiopia [28]. As a result a collaborative effort is needed to address the quality of life of people with depression through the integration of a bio-psycho social view in the pharmacological treatment approaches of depression [17, 27].

In Ethiopia, there is no available evidence reporting about the health related quality of life of people with depression, and little is done regarding the positive mental health approach and psychological construct of people with severe mental illness. Therefore, the current study was aimed to fill this gap by assessing the health related quality of life and its predictors among people with depression in Ethiopia. The findings of this study can be important for clinicians, social workers, psychologist, planners, decision makers and mangers. Furthermore, it can be used as baseline information for further studies.

## Methods

### Study design and period

This was an institutional based cross sectional study conducted from May ^1st to 30th^, 2018.

### Study setting and participants

The study was conducted at Amanuel Mental Specialized Hospital (AMSH). AMSH is the first hospital started mental health service in Ethiopia since its establishment at 1930 [28]. Currently, AMSH is the only specialized psychiatric hospital in the country serving for people from the nine regions and two city administrations of the country. The hospital provides service for people with psychiatric, neurological, substance and psychosocial problems in both outpatient and inpatient cares. The hospital has 259 beds for people with severe mental illness and emergency mental health conditions to be treated at inpatient services. AMSH hospital also has 18 different and separate ODPs serving for 7, 442 monthly estimated individuals with different diagnostic case teams (psychotic, mood, forensic, neurologic, substance, geriatric, NPS). Depression is the first rank based on the number of clients estimated to be served by the hospital every month. The diagnostic level of clients is confirmed by senior psychiatrists of AMSH at the first visit and their clinic condition is also evaluated during the follow up visit. The next follow up visit of clients is scheduled first and documented in the registration book of the hospital. Clients with age above or equal to 18 years, having follow-up visit for at leat 6 months, and having full insight regarding their illness and importance of their treatment were considered as eligible candidate to participate in the interview.

### Sampling and data collection techniques

The minimum number of samples required for the study was calculated by using single population mean formula with assumptions of 1.96 Z value at (∞ = 0.05), 9.65 standard deviation of the mean quality of life score from a study in Nigeria [10] and margin of error of 1. By considering a 10% non-response rate, the total sample size was 394.

Prior to the actual data collection, 2114 individuals with depression were expected to visit the hospital from May ^1st to 30th^ according to the schedule documented in the registration book of the hospital. Then, a sampling interval (K) was calculated by diving the total number of depressed patients expected to come to AMSH during the data collection time to the calculated sample size (K = 2114/394 = 5.37}. Then, eligible clients were selected for every six^th^ intervals according to the order of their follow up evaluation until the calculated sample size (394) was addressed.

The interview was conducted at a separate and private interviewing room adjusted for this purpose around the waiting hall of the hospital. The data collection facilitators sent eligible respondents to the prepared interviewing room and data collectors conducted the interview after obtain written consent. Six data collectors (BSc level psychiatric nurses) and two supervisors (MSc level mental health professional) were participated in the data collection after attending 2 days of training regarding the contents of questionnaire and data collection procedures. Finally, a total of 394 client invited to participate in the interview and 387 (98%) completed the interview properly.

### Data collection instruments

An interviewer administered questionnaire was used for the data collection. The questionnaire had different domains, including socio-demographic profile, world health organization quality of life brief (WHOQOL-BRFE), Oslo Social Support Scale, Morisky Green Levine Medication Adherence Scale, Jacoby Stigma Scale, clinical related characteristics and questions related substance use.

The outcome variable (quality of life) was measured using the WHOQOL-BRFE. The tool had 26 items measuring the physical health, psychological state, social relation, and environment aspects. In the physical health domain of the WHOQOL-BRFE, sleep pattern, working capacities, energy and medications use were assessed. In the psychological domain, we measured the thinking, body image and spiritual aspects where as relationships and sexual relations were addressed with in social domain. Finally, the environmental domain addressed the safety, leisure, finance, home and information aspects. WHOQOL-BRFE is a cross-culturally validated instrument to measure the quality of life, particularly useful when addressing the impact of physical and psychological well-being, but also on several domains beyond health, and had good sensitivity and specificity to assess the quality of life of people at health care settings [29]. We have used the Ethiopian validated Version of WHOQOL-HIV-BREF-Eth with good acceptability and psychometric properties [30]. The Cronbach alpha of WHOQOL-HIV-BREF-Eth was 0.82 in this study. QOL raw scores are transformed in to a range between 0 and 100. Scores are scaled in a positive direction (i.e. higher scores correspond to a better health related quality of life and vice versa [31].

Data for independent variables were also collected using standardized tools. Accordingly, Oslo Social Support Scale was used to measure the social support level of study participants. OSSS-3 has been recommended to be used for epidemiological and population-based surveys. The tool has three questions used to assess the numbers of people so close to be counted during great personal problems, level of interest and concern do people show and the accessibility of practical help from neighbors if needed, respectively. The first question has four response options ranging from 1 (none) to 4 (more than five) while the second and third questions has five options. The sum score of OSSS-3 ranges from 3 to 14. The higher value of the sum score represents stronger levels of social support and vice versa. Based on the sum score of OSSS-3, the level of social support is also categorized into three levels (poor = “3–8”, moderate = “9–11”, strong = “12–14”) [32]. The tool has been used in different studies with acceptable sensitivity and specificity. In the current study, OSSS-3 showed a good internal consistency having a Cronbach alpha of 0.91.

Morrisky Green Levine Medication Adherence Scale was used to evaluate psychotropic medication adherence level of people with depression. The tool had four different “Yes” or “No” questions scored for 1 and 0, respectively and individuals with a sum score of four and above were considered as having poor medication adherence [33].

Regarding the assessment of perceived stigma, we used the Jacoby Stigma Scale with three item questions to assess the individual’s perception of stigma regarding their illness. Each of the three questions had two possible responses and scored 0 for “no” responses and 1 for “yes” responses. The tool had a sum scores ranging from 0 to 3 where a score of 1 and above indicating that the patient is stigmatized [34]. The Jacoby Stigma Scale had a Cronbach alpha of 0.7 in the current study. Current substance was also measured by directly asking the client “Yes” or “No” question whether he/she had used substance in the last 1 month or not.

The questionnaire was originally prepared in English and translated into the local language (Amharic) by senior English language experts who are fluent of the local language, and back translated to English by other persons with similar profession and educational level to check its consistency. The questionnaire was pretested at St. Paul’s hospital among 5% of the calculated sample.

### Data analysis and interpretation

The collected data were checked for its completeness and consistency. Then, the data were entered to EPi-DATA version 3.1 and transformed to SPSS version 20 for analysis. First, the correlation of each variable was checked and their correlation with health related quality of life using linear regression. Then, variables with *P*-value of less than 0.25 were entered together to the multiple linear regression analysis. Accordingly, age of clients, sex, age of onset of depression, residency, marital status, perceived stigma, living arrangement, level of social support, substance use and follow up duration were entered to multiple linear regression. In the multiple linear regression analysis, age of respondents, age of onset of depression, perceived stigma, living arrangement, social support level and duration of illness were found as a statistically significant predictors of health related quality of life with *P*-values of less than 0.05. The strength of the correlation was also measured by β coefficient with corresponding 95% CI.

## Results

### Socio-demographic characteristics of respondents

Out of 394 study participants invited to be participated in the interview, 387 completed the interview properly with a response rate of 98%. The mean standard deviation (+SD) age of respondents was 40 ± 8 years. More than half, (58.4%) of respondents were males, and 80.6% reside in urban areas. Majority, (54%) of participants were married and living together. The mean scores of perceived stigma and social support level were 0.6 and 9.1, respectively (Table 1).

**Table 1:** Socio-demographic and psycho-social characteristics of people with depression attending outpatient department at AMSH, Addis Ababa, Ethiopia, 2018 (*n* = 387)

Variables	Categories	Frequency	Percentage
Age of respondents (Mean ± SD)40 ± 8			
Sex	Male	226	58.4
	Female	161	41.6
Ethnicity	Amhara	180	46.5
	Oromo	160	41.3
	Tigre	30	7.8
	Others^a^	17	4.4
Religion	Orthodox	201	51.9
	Muslim	103	26.6
	Protestant	70	18.1
	Others^b^	13	3.4
Residence	Urban	312	80.6
	Rural	75	19.4
Marital status	Single	209	54.0
	Married	99	25.6
	Divorced	60	15.5
	widowed and separated	19	4.9
Educational status	unable to read and write	24	6.2
	primary education	110	28.4
	secondary education	73	18.9
	Diploma, degree and above	180	46.5
Work status	Private business	141	36.4
	Jobless	150	38.8
	Employed	79	20.4
	Others^c^	17	4.3
Living arrangement	With families	280	72.4
	Alone	107	27.6
Social support	Strong	46	11.9
	Moderate	185	47.8
	Poor	156	40.3
Perceived stigma	Yes	245	63.3
	No	142	36.7

*Abbreviation*: *AMSH* Amanuel Mental Specialized Hospital

^a^Gurage, wolayta, Somaliee, Afar, ^b^Catholic, wakefeta, Hawariyat, ^c^Farmer, Student

### Clinical and substance related characteristics

The median age of onset of depression was 32.0 years. About 67(17.3%) of participants had an additional co-morbid medical illness (Diabetes mellitus (5.1%), cardiac disease (3.4%), HIV/AIDS (2.2%), epilepsy (4.3%) and others (2.2%). About 30.3% of participants were current substance users (Table 2).

**Table 2:** Clinical related characteristics of people with depression attending outpatient department at AMSH, Addis Ababa, Ethiopia, 2018(*n* = 387)

Variables	Categories	Frequency	Percentage
Duration of the illness	Less than 5 years	219	56.6
	5–10 years	100	25.8
	Greater than 10 years	68	17.6
Co morbid medical diagnosis	No	320	82.7
	Yes	67	17.3
Medication adherence	Adherent	191	49.4
	Non- adherent	196	50.6
Lifetime substance use	Yes	321	82.9
	No	66	17.1
Current substance use	Yes	117	30.2
	No	270	69.8

*Abbreviation*: *AMSH* Amanuel Mental Specialized Hospital

### Quality of life of people with depression

Nearly half of the study participants scored below the mean score of the WHOQOL-BRIEF quality of life for all the physical, psychological, social and environmental domains (Table 3).

**Table 3:** Distributions of quality of life domains among people with depression attending outpatient department at AMSH, Addis Ababa, Ethiopia, 2018 (*n* = 387)

Domains	Maximum	Minimum	Mean ± SD	Scores below mean	95%CI
Physical	53.57	25.00	41.3 ± 7.5	43.9%	(40.62, 42.14)
Psychological	58.33	20.83	42.8 ± 8.2	52.7%	(41.94, 43.61)
Social	58.33	25.00	38.9. ± 8.7	44.7%	(38.01, 39.84)
Environmental	53.13	28.13	41.8 ± 6.5	51.4%	(41.12, 42.43)

*Abbreviations*: *AMSH* Amanuel Mental Specialized Hospital, *SD* standard deviation

### Correlates of quality of life of people with depression

The multiple linear regression showed that age of respondents, age of onset of depression, perceived stigma, living arrangement, social support level and duration of illness were found as a statistically significant correlates of health related quality of life for atleast one domain (Table 4).

**Table 4:** Multiple linear regression models for physical, psychological, social and environmental domains of quality of life among people with depression attending outpatient department at AMSH, Addis Ababa, Ethiopia, 2018 (*n* = 387)

Variables		Domains of health related quality of life			
		Physical	Psychological	Social	Environmental
		B Coefficient with 95% CI	B Coefficient with 95% CI	B Coefficient with 95% CI	B Coefficient with 95% CI
Age of participants		0.34 (0.27, 0.42)***	0.37 (0.27, 0.47)***	0.53 (0.42, 0.64)***	0.44 (0.36, 0.52)***
Age of onset of depression		0.26 (0.20, 0.33)***	0.27 (0.19, 0.36)**	0.07 (−0.02, 0.17)	−0.09 (− 0.07, 0.05)
Sex	Male	Ref	Ref	Ref	Ref
	Female	0.03 (−0.91, 0.99)	0.53 (−0.67, 1.75)	0.57 (− 0.75, 1.90)	0.28 (− 0.70, 1.26)
Residency	Urban	Ref	Ref	Ref	Ref
	Rural	1.02 (−0.53, 2.58)	−0.16 (2.13, 1.80)	0.903 (−1.31, 3.1)	− 0.98 (− 2.56,.59)
Marital status	Married	Ref	Ref	Ref	Ref
	Single	−0.31 (−1.38, 0.74)	0.515 (− 0.81, 1.8)	1.15 (− 0.40, 2.70)	−0.84 (1.96, 0.27)
	Divorced/widowed	−1.11 (− 2.35, 0.12)	− 1.4 (− 3.18, 0.2)	− 0.44 (− 2.15, 1.26)	−1.58 (− 2.8, 0.3)
Perceived stigma	No	Ref	Ref	Ref	Ref
	Yes	−2.38 (−3.38, −1.3)**	−1.02 (− 2.33, 0.29)	0.74 (− 0.82, 2.31)	0.38 (− 0.67, 1.44)
Living with	Families	Ref	Ref	Ref	Ref
	Alone	-1.92(-3.09,-0.74)*	0.41 (− 0.92, 1.76)	− 1.27 (− 2.79, 0.24)	− 0.52 (− 1.61, 0.57)
Social support level	Strong	Ref	Ref	Ref	Ref
	Medium	0.55 (− 0.42, 1.52)	−1.29(−2.74, 0.14)	− 0.30 (− 2.07,	−0.25 (− 1.21
	Poor	−0.65 (− 1.71, 0.41)	−4.8 (− 6.4, −3.3)***	− 3.78 (− 5.3, −2.2)***	−3.4 (− 4.6, −11.34)***
Substance use	No	Ref	Ref	Ref	Ref
	Yes	− 1.19 (− 2.43, 0.05)	− 0.11 (− 1.56, 1.34)	−1.29 (− 2.87, 0.28)	0.79 (− 0.38, 1.96)
Duration of depression	< 5 years	Ref	Ref	Ref	Ref
	5–10 years	0.02 (−1.06, 1.07)	0.01 (−1.32, 1.34)	−3.09 (− 4.61, 1.59)*	− 2.20 (− 3.41, −1.13)***
	> 10 years	-1.67(-2.9,-0.4)***	− 0.73 (− 2.28, 0.81)	− 0.46 (− 2.24, 1.29)	− 0.58 (− 1.83, 0.67)

*Abbreviation*: *AMSH* Amanuel Mental Specialized Hospital

*P < 0.001, ** *P* < 0.01, ***P < 0.05

## Discussion

A new and innovative clinically based evaluation method has been introduced for the assessment of levels of quality of life and psychological distress in patients with depression [22]. Clinicians are supposed to use these newly emerging innovations as a treatment modality of people with depression [23].

However, literatures conclude that the health related quality of life of people with depression is not still well addressed in developing nations including Ethiopia [17, 20, 28]. The findings of the current study also supported this conclusion by showing that nearly half of the study participants scored below the mean score of health related quality of life for all domains. The mean scores of health related quality of life of people with depression in Ethiopia were 41.3, 42.8, 38.9, and 41.8 for physical, psychological, social and environmental domains, respectively. These results are supported by the conclusions of a previous study of Brazil [35], South Africa [36] and Germany [37] which also concluded that health related quality of life of people with depression is very poor. The, nature of symptoms, its comorbod illnesses, impaired self image, social, occupational and cognitive impairments and poor social relationship can play a great role for the lowered quality of life of people with depression [11, 16].

However, a study conducted in Pakistan reported that majority of patients with major depressive disorder had a poor QOL with a mean score of 0.26 ± 0.3 [7]. This much lower than the mean scores of all domains of health related quality of life reported in the current study. The explanation for the discrepancy of the results might be due to the socio-cultural variations of study participates and the differences of the screening tools used to measure quality of life [30].

A study from China reported a better mean score of quality of life of outpatients with depression in each domain of health related quality of life [38]. Another cross sectional study in Nigeria among patient with schizophrenia and major affective disorders (*n* = 108) 2 weeks after discharge using WHOQOL BREF also showed a better quality of life; at least two-thirds of the subjects were categorized as having better (average) QOL in the physical, psychological and environmental domains of QOL [39]. This is higher than the finding of the current study regarding the quality of life of people with depression. The discrepancy of these results might be due the better availabilities of strong health care system and shorter duration of the illness in China as compared to Ethiopia [38]. Moreover, this might be due to the large sample size (*n* = 1,984) used, the difference in the socio-demographic characteristics of participants, and also the involvement of only first visit patients as participants in a study conducted [8].

The current study was also aimed to identify predictors of health related quality of life of people with depression attending outpatient department in Ethiopia. Accordingly, age of respondents had positive correlation with all domains of health related quality of life. As age of respondents increased by 1 year their health related quality of life increases by 0.34, 0.37, 0.53, and 0.44 units for physical, psychological, social and environmental domains, respectively. This finding is supported by a study of three European countries [40] i.e. the younger the age of study respondents was a predictor for the poorer the quality of life of their lives. This might be due to the higher tendency to come into a state of acceptance towards themselves and their lives as people become older [36].

Patients with early onset of depression and longer follow up duration (greater than 5 years) had a markedly diminished perception of their health related QOL. This finding is in line with a previous study conducted among outpatients with depression in China [41]. This might be explained by the fact that patients with early onset of depression and longer follow up duration are more likely to have an unfavorable prognosis, higher rates of relapse, chronicity of the illness which increases the likelihood of dissatisfaction with their health related QOL [37].

The physical health domain of QOL was decreased by 2.38 units among study participants with perceived stigma because of their depressive status. This is consistent with the conclusions of other similar studies done in Jordan [14], Czech [42] and Taiwan [11]. This is possibly explained by the lowered self-esteem, social isolation and poor community engagement of people with perceived stigma of their illness with might lead to less satisfaction in their quality of life [42]. The social support level was positively correlated with health related QOL of people with depression. Patients with a stronger social support level had a better health related QOL except the physical domain. This finding is supported by other studies of Argentina [10], Germany [37] and China [41]. This might be due to the fact that people with stronger social support can have a better self image, social relationships, safety and engagement to the community [43]. Furthermore, individuals with stronger social support commonly share moments of both their happiness and distress with someone close to them and get relief from their distress [44].

Similarly, the physical health domain of QOL had reduced by 1.92 units among patients with depression who were living alone as compared to those living with their own families. This is in agreement with a study from Sweden which revealed that living with families was related to a better quality of life [15]. This might be due to the fact that living alone by itself can have more psychological distress and worsen the lowered self-image of people with depressive state [43].

In general, the findings of the current study recommends for professionals providing service for people with mental illness to improve the quality of life of people with depression by integrating the newly innovated positive mental health approach and psychosocial support together with the pharmacological treatment of depression. Health managers and policy makers are also expected to consider this issue in their plan, and appropriate resources should be allocated.

### Strength, limitations and generalisability of the study

This study was conducted using standardized cross-culturally validated tools to measure quality of life and other variables. Additionally, appropriate translation of the questionnaire and proper supervisition of the data collection were done. For the generalisability of the study, a probability sampling technique was used. However, the cross-sectional nature of the study design might not show the cause and effect relationship between variables and conducting further follow-up studies is highly recommended.

## Conclusion

Nearly half of the study participants scored below the mean score of the WHOQOL-BRIEF quality of life for all the physical, psychological, social and environmental domains. Age of respondents, age of onset of depression, perceived stigma, living arrangement, social support level and duration of illness were statistically significant predictors of health related quality of life. This demonstrates a need for improving the quality of life of people with depression by integration of a positive mental health approach and bio-psychosocial view with the pharmacological treatments of depression. Moreover, strengthening social support, early identification and treatment of depression and prevention of stigma among individuals with depression is also highly recommended to improve the quality of life of people with depression.

## Data Availability

All the data included in the manuscript can be accessed from the corresponding author Seid Shumye upon request through the email address “seidshumye22@gmail.com”.

## Abbreviations

AMSH: Amanuel Mental Specialized Hospital
BD: Bipolar Disorder
BSc: Bachelor of Science
CI: Confidence Interval
CMD: Common Mental Disorder
CMHS: College Of Medicine and Health Science
DSM: Diagnostic and Statistical Manual of American Psychiatric Association
ETB: Ethiopian Birr
HRQOL: Health Related Quality Of Life
MDD: Major Depressive Disorder
MSc: Master of Science
OPD: Outpatient Department
OSSS: Oslo Social Support Scale
QOL: Quality Of Life
SD: Standard Deviation
SPSS: Statistical Package for Social Science
SQOL: Subjective Quality Of Life
WHOQOL-BREF: World Health Organization Quality of Life Brief Version
